# The Role of Flavonoids from Aurantii Fructus Immaturus in the Alleviation of Allergic Asthma: Theoretical and Practical Insights

**DOI:** 10.3390/ijms252413587

**Published:** 2024-12-19

**Authors:** Jingwen Xue, Yuntong Liu, Qiushi Chen, Huimin Liu, Huijing Zhang, Bo Wang, Yongri Jin, Xuwen Li, Xiaolei Shi

**Affiliations:** 1 College of Food Science and Engineering, Jilin University, Changchun 130062, China; byang@jlu.edu.cn (J.X.); zyp@jlu.edu.cn (Q.C.); ywz@jlu.edu.cn (H.L.); 2 College of Chemistry, Jilin University, Changchun 130012, China; yuntong20@mails.jlu.edu.cn (Y.L.); ybmol@163.com (H.Z.); bwang_chem@jlu.edu.cn (B.W.); jinyr@jlu.edu.cn (Y.J.); xwli@jlu.edu.cn (X.L.)

**Keywords:** hypersensitivity, computational biology, allergy and immunology

## Abstract

Flavonoids derived from plants in the citrus family can have an alleviating effect on allergic asthma. The aim of this study was to provide insights into the mechanisms by which these compounds exert their effects on allergic asthma by combining theoretical and practical approaches. Aurantii Fructus Immaturus flavonoids (AFIFs) were obtained by solvent extraction and were determined by high performance liquid chromatography (HPLC). In vivo and in vitro experiments combined with network pharmacology, Mendelian randomization (MR) analysis and the AutoDock method were applied to study the mechanism of their effects. The main AFIFs were found to be hesperidin (13.21 mg/g), neohesperidin (287.26 mg/g), naringin (322.56 mg/g), and narirutin (19.35 mg/g). Based on the network pharmacology and MR analysis results, five targets Caspase 3 (CASP3), CyclinD1 (CCND1), Intercellular adhesion molecule (ICAM), erb-b2 receptor tyrosine kinase 2 (ERBB2), and rubisco accumulation factor 1 (RAF1) were selected, and the interactions between the AFIFs and the targets were studied using AutoDock Vina. The results indicated that glycosidic bonds play an important role in the interactions between AFIFs and both ERBB2 and RAF1.

## 1. Introduction

Aurantii Fructus Immaturus (AFI) is the dried and unripe fruit of *Citrus aurantium* L. It is obtained by collecting self-falling fruit from May to June. This fruit has a black-green or brown outer skin after drying. It is considered a homologous food and medicine in China. Clinically, AFI is used for gastrointestinal food retention, fullness and pain from distention of the stomach, and prolapse of the rectum and uterus [1]. Flavonoids are the main ingredients of AFI (AFIFs) [2]. According to references, high dietary flavonoid intook may be associated with a low risk of chronic bronchitis and asthma in males in the U.S. population [3]. In addition, a diverse diet rich in flavonoids could benefit women with asthma by mitigating inflammation and oxidative stress [4]. Research has demonstrated that hesperidin, narirutin, naringin, and neohesperidin are the main AFIFs. 

Allergic asthma is defined as asthma associated with hypersensitization to aeroallergens. It is characterized by asthma symptoms and airway inflammation. Allergic asthma is the most common asthma phenotype [5]. The onset of allergic asthma most commonly occurs in childhood and is often accompanied by atopic dermatitis and allergic rhinitis. Allergic asthma is considered a Th2-driven process, with patients exhibiting high serum IgE levels. This condition not only affects the health and quality of life of patients but also poses an economic burden due to the high cost of medical treatment [6]. Thus, there is a critical need to explore cost-effective and efficacious treatments for allergic asthma. To date, researchers have tested various approaches to achieve effective treatment of allergic asthma, such as blocking a single mediator of asthma, but the clinical effects have not been satisfactory [7]. Multi-target drugs capable of inhibiting multiple critical activation pathways are likely to be more efficacious in complex diseases like severe asthma [8]. Studies have indicated that natural products can be efficacious in the clinical treatment of allergic asthma [9], and these products have several advantages, such as low costs and few side effects. The most important feature of these products is their multiple targets. The multi-target characteristics of natural products with complex compositions may confer better bioactivity. However, there is a scarcity of research addressing this topic, as it is difficult to study the mechanisms of complex compositions.

Randomized controlled trials (RCTs) are the gold standard for determining a compound’s efficacy. Products with complex compositions and multiple targets require significant investments of money and effort. With the development of genome-wide association studies (GWASs), Mendelian randomization (MR) analysis has emerged as an effective method to solve this problem [10]. The expression or function of target genes can be affected by genetic variations within genes encoding protein targets. This is similar to the mechanism of active drugs. 

The prerequisite for conducting studies such as the current study is the identification of the targets of drugs. This is not difficult for known drugs but poses a huge challenge for mixtures of unknown complex components [11]. Network pharmacology is an effective method to uncover the complex mechanisms underlying products with multiple components and multiple targets [12]. In this work, network pharmacology was applied to explore the targets of AFIFs, and MR analysis was used to verify the causal relationships between exposure factors (AFIFs) and allergic asthma using genetic variation as an instrumental variable (IV).

The aim of the present work is to clarify the role of flavonoids (hesperidin, neohesperidin, naringin, and narirutin) from AFI in the alleviation of allergic asthma by theoretical and practical methods. First, AFIFs were extracted using the solvent reflux method. The main AFIFs were found to be hesperidin, neohesperidin, naringin, and narirutin. Then, in vivo and in vitro experiments were performed to evaluate the effects of AFIFs on allergic asthma. The purpose was to clarify the specific inhibitory effects of AFI on allergic asthma. Second, a comprehensive network pharmacology analysis was conducted to explore the core targets of AFIFs and study the potential mechanism of AFIFs on allergic asthma. Third, MR analysis was applied to study the causal relationships between AFIFs and allergic asthma via the core targets. Finally, structural analysis between flavonoids (similar structures) and core targets was performed using AutoDock Vina. The ultimate goal of this work was to validate and extend the application of AFIFs and provide insight into the mechanism by which these compounds exert their alleviating effect on allergic asthma. This study also highlights the utility of combining theoretical and practical approaches to explore the mechanisms underlying the physiological activity of complex mixtures with multiple targets.

## 2. Results

### 2.1. Composition of AFIFs

**Figure 1:**
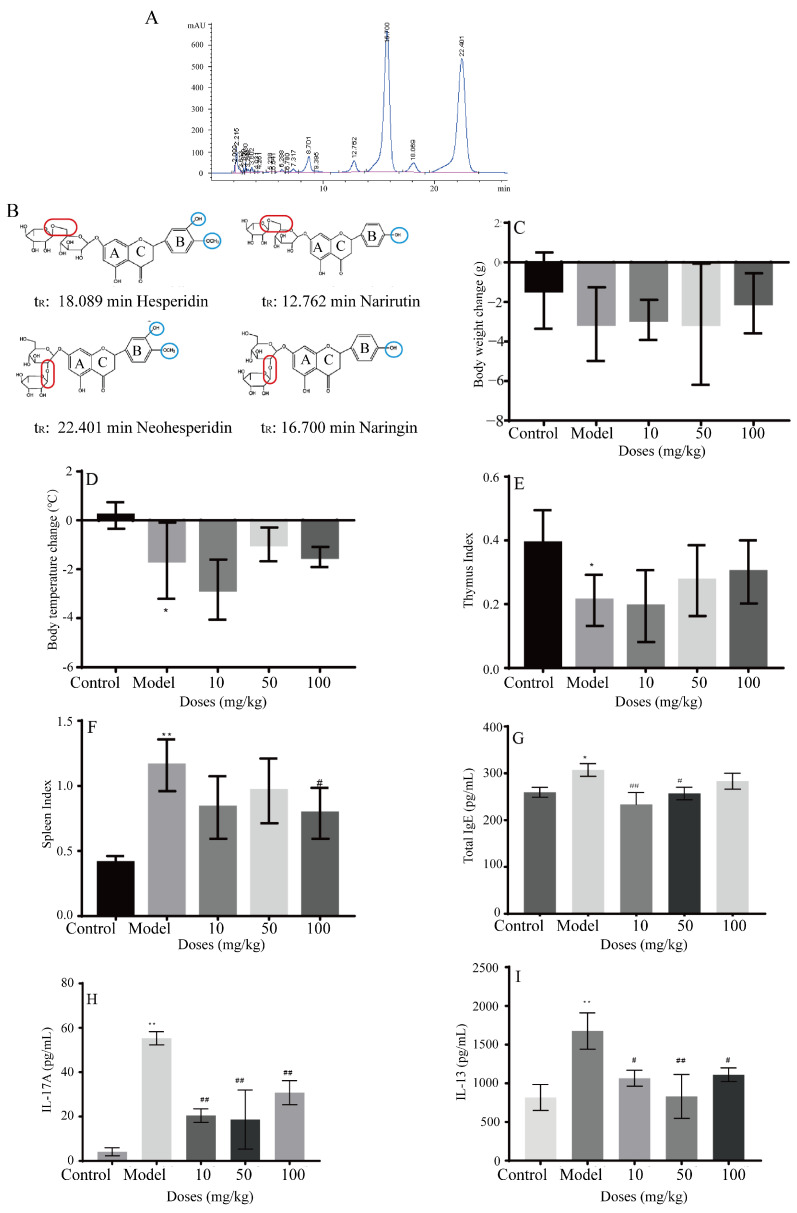
HPLC analysis of AFIFs and effects of AFIFs on body weights, rectal temperature, spleen, thymus index and inflammatory response in vivo. (**A**) HPLC of AFIFs; (**B**) Structures of hesperidin, neohesperidin, naringin, and narirutin. Red circle presents the different connection of glycosidic bonds; Blue circle presents the different connection of -OH and -OCH_3_; (**C**) Effects of AFIFs on body weight changes; (**D**) Effects of AFIFs on rectal temperature change; (**E**) Effects of AFIFs on thymus index; (**F**) Effects of AFIFs on spleen index; (**G**) Effects of AFIFs on total IgE; (**H**) Effects of AFIFs on IL-17A in BALF; (**I**) Effects of AFIFs on IL-13 in BALF. * *p* < 0.05, ** *p* < 0.01, compared with the Control group; # *p* < 0.05, ## *p* < 0.01, compared with the Model group.

The concentrations of hesperidin, neohesperidin, naringin, and narirutin in AFIFs were 13.21, 287.26, 322.56, and 19.35 mg/g, respectively (Figure 1A). As shown in Figure 1B, the structures of flavonoids were similar. The differences between hesperidin /neohesperidin and narirutin/naringins were observed on the B part of the flavonoids. The 3′-C of hesperidin/neohesperidin connected with OH, but this connection was not observed with narirutin/naringin. Moreover, the 4′C of hesperidin/neohesperidin is connected to OCH_3_, but OH is connected to narirutin/naringin on the 4′-C.

### 2.2. Effects of AFIFs on Allergic Asthma In Vivo

#### 2.2.1. Effects of AFIFs on Body Weight, Rectal Temperature, and Organ Coefficients

From days 1–14, the body weights of all groups were increased. However, from days 14 to 24, the trends in the groups differed. The body weight change (BWC) results are shown in Figure 1C. From days 14 to 24, the BWCs in the control group were slightly decreased. Compared with the control group, the BWCs in the model group were significantly decreased. This decrease was inhibited in the AFIFs groups. The rectal temperature results are shown in Figure 1D. The 50 and 100 mg/kg AFIFs groups exhibited inhibition of the reduced rectal temperature induced by OVA, but this difference was not statistically significant. The thymus and spleen indices are shown in Figure 1E F. For the spleen index, 10 and 100 mg/kg of AFIFs significantly inhibited the increase in the spleen index induced by OVA. There were no effects of AFIFs on the thymus index.

#### 2.2.2. AFIFs Alleviated Lung Tissue Damage and the Inflammatory Response In Vivo

A high level of serum IgE is a typical characteristic of allergic asthma and is closely related to increased Th2 cytokines. The serum concentrations of IgE in the different groups of mice were analyzed (Figure 1G). The IgE level in the model group was significantly higher than the control group (*p* < 0.01). Significantly decreased IgE concentrations were observed in the 10 mg/kg (*p* < 0.01) and 50 mg/kg (*p* < 0.05) AFIFs groups. These results indicate that AFIFs reduced the total IgE levels in serum. 

IL-17A in bronchoalveolar lavage fluid (BALF) (Figure 1H) is a pro-inflammatory factor produced by Th17 cells. It plays a key role in the innate and adaptive immune response against respiratory bacterial colonization and infection. IL-17A was decreased by the different doses of AFIFs.

The concentrations of Th2 cytokines, such as IL-13 in BALF, were then investigated (Figure 1I). The infiltration of lung tissues is strongly related to Th2 cytokine release. The level of IL-13 (*p* < 0.01) in the model group was significantly increased compared with the control group and was decreased by AFIFs at doses of 10, 50, and 100 mg/kg (*p* < 0.01).

**Figure 2:**
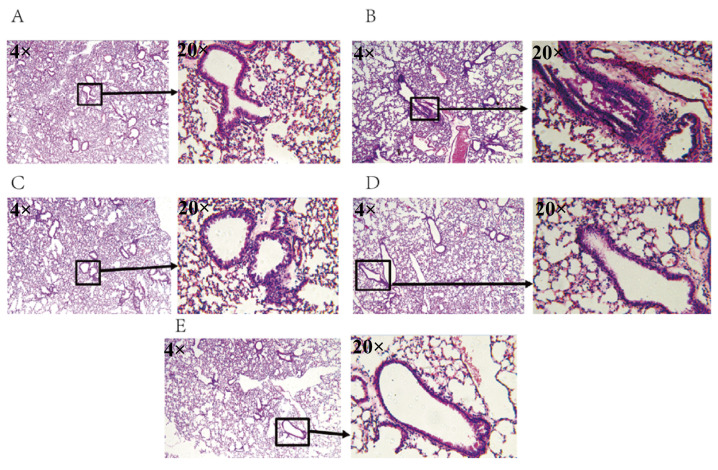
(**A**) Histomorphological changes in the lungs of mice (Control); (**B**) Histomorphological changes in the lungs of mice (Model); (**C**) Histomorphological changes in the lungs of mice (AFIFs dose 10 mg/kg); (**D**) Histomorphological changes in the lungs of mice (AFIFs dose 50 mg/kg); (**E**) Histomorphological changes in the lungs of mice (AFIFs dose 100 mg/kg).

To investigate the effects of AFIFs on lung tissue damage, H&E staining was applied (Figure 2A–E). As can be seen, in comparison to the control group, mice in the asthma group exhibited pathologic changes in the bronchial epithelium, eosinophils infiltration, thickening of the sub-epithelial smooth muscle layer, and hyperemia. The AFIFs groups exhibited decreased inflammation and other abnormalities. 

#### 2.2.3. Effects on RBL-2H3 Cell Degranulation In Vitro

**Figure 3:**
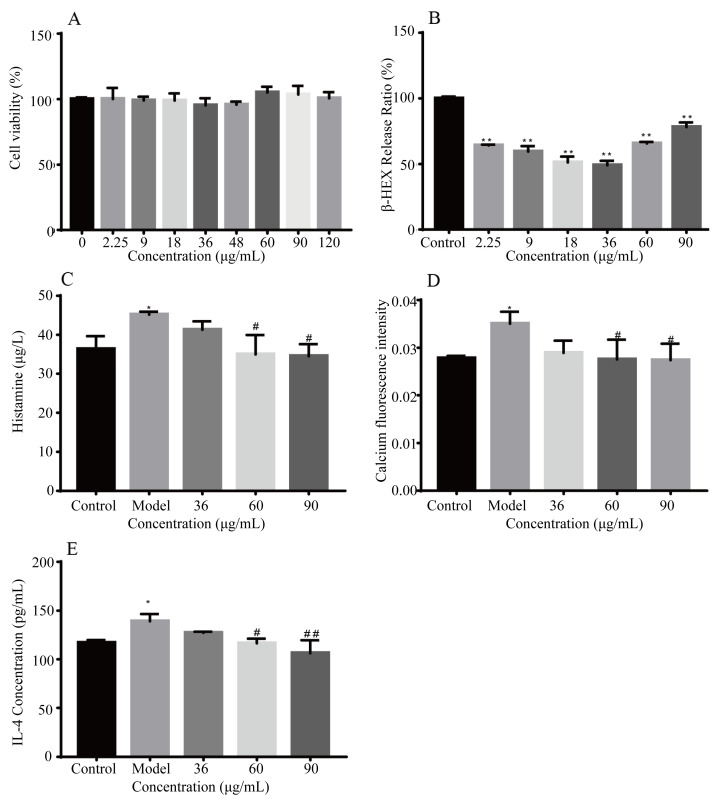
Effects of AFIFs on mast cell degranulation. (**A**) Effects of AFIFs on cell viability; (**B**) Effects of AFIFs on β-HEX; (**C**) Effects of AFIFs on Ca^2+^ influx; (**D**) Effects of AFIFs on IL-4 release; (**E**) Effects of AFIFs on histamine release. * *p* < 0.05,** *p* < 0.01, compared with the Control group; # *p* < 0.05, ## *p* < 0.01, compared with the Model group (0 μg/mL).

As shown in Figure 3A, no significant effects on cell viability were observed. As shown in Figure 3B,C,E, with increased doses of AFIFs, the β-hex, histamine, and the IL-4 release decreased significantly. The intracellular Ca^2+^ concentrations also decreased significantly with AFIF doses of 60 and 90 μg/mL (Figure 3D). 

### 2.3. Network Pharmacology of AFIFs

Hesperidin, neohesperidin, naringin, and narirutin were found to be the active flavonoids in the AFIF extracts by HPLC qualitative analysis. After eliminating overlapping targets, 17 targets were identified in the TCMSP database. These 17 targets were searched in the STRING database to identify more potential targets, with the following parameters: 1st shell ≤ 10 and 2nd shell ≤ 20. A total of 47 targets were obtained.

**Figure 4:**
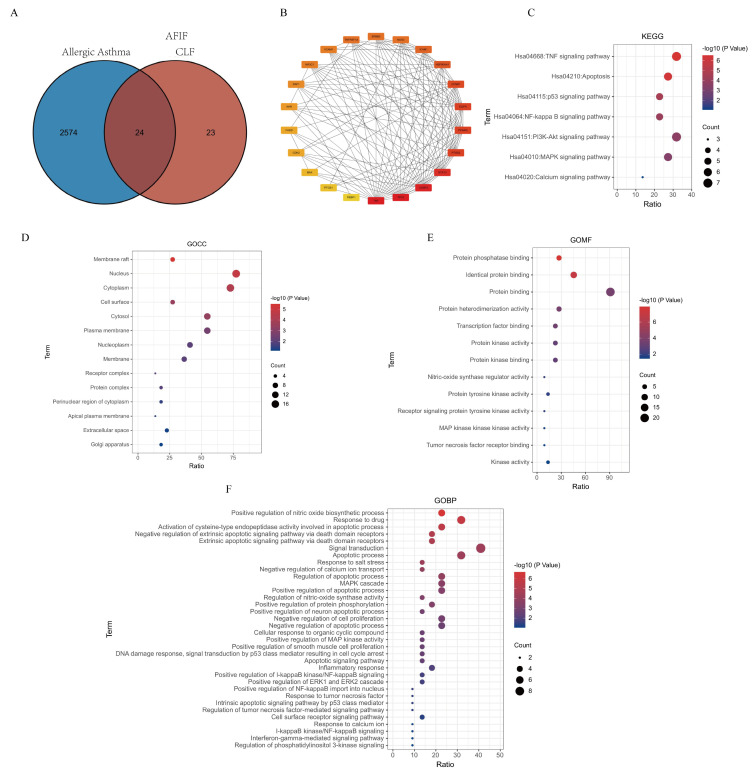
Network pharmacology of AFIFs and allergic asthma. (**A**) The number of allergic asthma-related targets and AFIFs targets showed by Venn; (**B**) Network of 22 targets; (**C**) KEGG analysis; (**D**) GO-CC analysis; (**E**) GO-MF analysis; (**F**) GO-BP analysis.

The targets for allergic asthma were integrated from GeneCards, and a final list of 2598 disease-related targets was obtained after the ‘above the median value’ parameter was set. A total of 24 overlapping targets were obtained, and 22 targets were identified as the key targets for studying the anti-allergic asthma activity of the flavonoids from AFIFs (Figure 4A). There were no interactions between CCNA2 and CDN1A and any other targets.

To clarify the molecular mechanism underlying the effects of AFIFs against allergic asthma, a PPI network was obtained. The 22 intersecting targets were input into the STRING database to analyze the correlations between them (Figure 4B). These targets may be the key therapeutic targets of AFIFs in the treatment of allergic asthma. 

To clarify the relevant pathways and functions, KEGG pathway enrichment and GO functional analysis were performed for the 22 targets using David. As shown in Figure 4C, the KEGG enrichment analysis indicated that the signaling pathways were notably enriched in pathways such as the TNF signaling pathway, apoptosis, PI3k-Akt, and MAPK. 

GO analysis of the 22 targets of the flavonoids in AFIFs for allergic asthma was performed in relation to three categories: Biological Process (BP), Molecular Function (MF), and Cellular Component (CC). A total of 189 enrichment results were obtained, including 130 BP, 24 CC, and 35 MF. The typical results are shown in Figure 4D–F, including 14 enrichment results for CC, 34 for BP, and 13 for MF. *p* < 0.5 was considered to indicate statistical significance; a lower *p*-value was taken to indicate greater relevance.

### 2.4. MR Analysis

**Figure 5:**
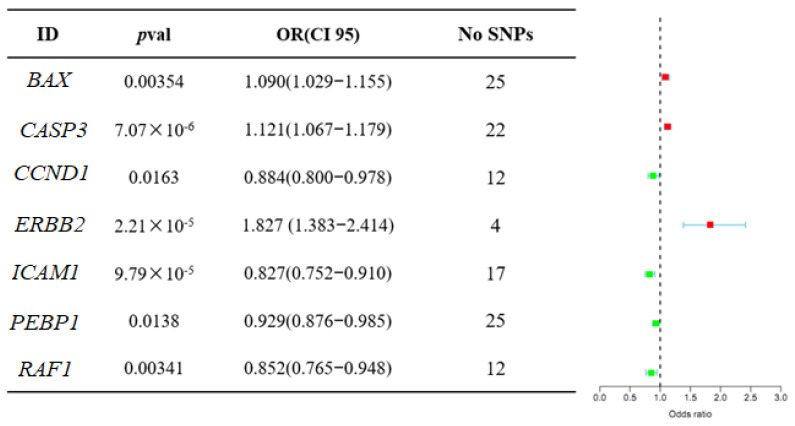
MR association between expression of gene *BAX*, *CASP3*, *CCND1*, *ERBB2*, *ICAM1*, *PEBP1*, *RAF1*, and allergic asthma outcomes. Red pots indicated Odds ratio > 1, Green pots indicated Odds ratio < 1.

The causal associations between AFIFs and allergic asthma were investigated using the MR approach. It was an effective method to generate effect estimates as eQTLs were applied as instruments. In the network pharmacology results, 22 targets were identified as AFIF targets. A total of 35 cis-eQTLs from the 22 targets were identified from eQTLGen. A total of 117 SNPs within or near genes, including *BAX*, *CASP3*, *CCND1*, *ERBB2*, *ICAM1*, *PEBP1*, and *RAF1*, were selected from the GWAS summary data. Based on the inverse variance weighted method, the MR analysis provided evidence of associations between allergic asthma and *BAX*, *CASP3*, *CCND1*, *ERBB2*, *ICAM1*, *PEBP1*, and *RAF1* in the blood. The odds ratios (ORs) are shown in Figure 5. 

To further evaluate the influence on allergic asthma, 117 independent SNPs were identified as instrumental variables. As can be seen in Table S2, the *F*-statistics of the SNPs were all >10 (ranging from 13.533 to 1970.019). The results indicated that all IVs had high instrumental strength. According to the MR-Egger results, the results were not affected by horizontal pleiotropy. According to the statistical values in Table S3, there was no heterogeneity between the independent SNPs, as demonstrated by Cochran’s Q test. The pleiotropy test returned *p*-values of the MR-Egger intercept that were greater than 0.05, suggesting no evidence of directional pleiotropy. However, the pleiotropy *p*-values of *BAX* and *PEBP1* were lower than 0.05, indicating that pleiotropy was present. The SNPs were then studied in detail using PS2. rs113360897, rs611251, rs11668424, rs1805419 of BAX, and rs2936839 of *PEBP1* exhibited pleiotropy, wherein rs113360897, rs611251, and rs2936839 were related to red cell distribution width and inflammation in patients with non-dipper hypertension. rs11668424 and rs1805419 were related to sum eosinophil basophil counts. Thus, these SNPs were deleted. 

### 2.5. Molecular Docking

**Figure 6:**
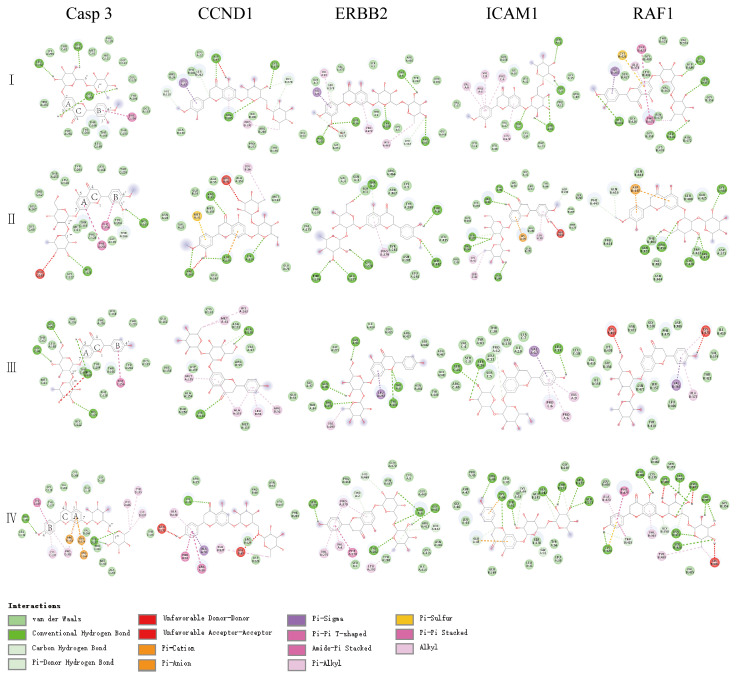
Molecular docking between key protein and AFIFs; color balls indicated amino acid residues of different targets. (**I**) interaction between hesperidin and key protein; (**II**) interaction between neohesperidin and key protein; (**III**) interaction between naringin and key protein; (**IV**) interaction between narirutin and key protein.

The four flavonoids in AFIFs were docked with CASP3, CCND1, ERBB2, ICAM1, and RAF1. The ligand and protein results are represented by a ball and stick and a carton chain, respectively. The results are shown in Figure 6. In general, the Vina score suggests a certain binding activity between a ligand and a protein. The stability of compounds bound to the protein is evaluated by a negative Vina score. The molecular docking results are shown in Table 1 and Table 2. The Vina scores of the four flavonoids binding with the targets were all negative and less than −5.0 kcal/mol. This indicates that hesperidin, neohesperidin, naringin, and narirutin exhibited good binding activities to the targets. 

## 3. Discussion

In recent years, AFI has attracted significant attention as a source of bioactive compounds. In Wang’s work, the total flavonoids from Qu Zhi Qiao exerted protective effects against OVA-induced allergic airway inflammation at doses of 50 and 100 mg/kg [13]. Therefore, in this study, the potential of AFIFs to alleviate allergic reactions was explored at low (10 mg/kg), medium (50 mg/kg), and high AFIF doses (100 mg/kg). 

Research has indicated that the mechanism underlying the allergic asthma mouse model induced by OVA is related to the Th2 cell response. IgE and IL-13 play major roles in the regulation of inflammation in allergic asthma. In the current study, AFIFs were found to down-regulate the IL-13 level in vivo, indicating that AFIFs can inhibit the Th2 response in the allergic asthma mouse model, thus decreasing the IgE level in the serum.

IL-17A is a pro-inflammatory cytokine produced by activated T cells. IL-17A mediates downstream pathways to induce the production of inflammatory molecules and chemokines. It stimulates non-hematopoietic cells and promotes chemokine production, thereby attracting myeloid cells to inflammatory sites. The current results demonstrated the inhibitory effect of AFIFs on chemokine production. The degranulation of mast cells is related to inflammation. Here, AFIFs were found to have a suppressive effect on mast cell degranulation.

Hesperidin is a flavonoid with anti-cancer and anti-inflammatory effects [14]. It can also protect against intestinal inflammation by restoring intestinal barrier function and upregulating Treg cells, suppressing OVA-induced airway inflammation [15]. Hesperidin inhibits the development of atopic dermatitis-like skin lesions by suppressing Th17 activity and the inactivation of NF-κB [16]. Neohesperidin suppresses IgE-mediated anaphylactic reactions and mast cell activation via the Lyn-PLC-Ca^2+^ pathway. Moreover, neohesperidin alleviates pathological damage and immunological imbalance via inactivation of JNK and NF-κB p65 [17]. Naringin can inhibit TNF-α/IFN-γ-induced RANTES expression via the NF-κB and p38 MAPK signaling pathways in vivo and in vitro [18]. Narirutin also inhibits airway inflammation in the allergic mouse model. Based on the current in vivo and in vitro results and the above literature, it can be concluded that AFIFs have inhibitory effects on allergic asthma. However, it is unreasonable to use the mechanism of action of a single compound to explain the mechanism of action of a mixture of compounds. Here, 22 targets were identified as key targets for studying the anti-allergic asthma activity of AFIFs. The current results indicated that the anti-inflammatory effects of AFIFs are mainly determined by these core targets.

Network pharmacology was used to explore the interaction targets of AFIFs. However, further investigation was needed to determine whether there are direct causal relationships between the core targets and allergic asthma. The present MR results suggested the presence of associations between the risk of allergic asthma and *BAX*, *CASP3*, *CCND1*, *ICAM1*, *PEBP1*, *RAF1*, and *ERBB2* expression. rs113360897, rs611251, rs11668424, and rs1805419 of BAX and rs2936839 of PEBP1 exhibited pleiotropy.

*CASP3* is an enzyme that executes cell apoptosis; it plays a role in airway smooth muscle layer thickening to induce airway narrowing during asthma attacks [19]. A functional exonic variant of ERBB2 has been shown to play a role in asthma development by modulating the MAPK signaling cascade [20]. *CCND1* is closely correlated with the development of asthma; in particular, *CCND1* rs9344 is considered an early detection marker for asthma [21]. *ICAM1* plays an important role in the inflammatory response, with some SNPs of *ICAM1* found to be associated with asthma [22,23]. *RAF-1* is a serine/threonine protein kinase that plays an essential role in airway smooth muscle cell proliferation [24].

MR is an effective approach for overcoming the limitations of traditional observational studies. In this work, core targets were obtained from Network pharmacology as instruments to proxy the exposure of AFIFs. The use of genetic instruments instead of drug exposure can minimize confounding bias and avoid reverse causation. The current results suggested that *CASP3*, *CCND1*, *ICAM1*, *RAF1*, and *ERBB2* expression are causally related to the risk of allergic asthma. 

Five targets with causal associations with the risk of allergic asthma were selected to analyze the interactions between proteins and the four flavonoids. The Vina scores indicated that each of the four flavonoids had good binding affinity to the five targets, and the structural differences did not have any impact on the affinity of the interactions with CASP3, CCND1, and ICAM. Due to the similarity of the interactions between AFIFs and the three proteins, the obtained Vina scores exhibited little variation. However, for the interaction between ERBB2 and RAF1, the tiny structural differences produce different effects. For hesperidin and neohesperidin, the structural differences were focused on the connection positions of glycosidic bonds. Hesperidin exhibited α-L-rhamnosyl-(1‴→6″)-β-D-glucopyranosyl and neohesperidin exhibited α-L-rhamnosyl-(1‴→2″)-β-D-glucopyranosyl. Compared with neohesperidin, hesperidin exhibited a stronger affinity with ERBB2. At the same time, narirutin exhibited a stronger affinity with ERBB2 than naringin, which had the same structural characteristics. Further, 3′C of hesperidin/neohesperidin connected with OH, 4′-C connected with OCH_3_, and 4′C of narirutin/naringin connected with OH; there were no affinity differences between hesperidin and narirutin (or neohesperidin and naringin). Moreover, hesperidin and narirutin had greater numbers of binding sites, amino acid residues interacting with targets, and amino acid residues interacting with glycosyl groups. This phenomenon was also observed in the interaction with RAF1. These findings suggest that the connection positions of glycosidic bonds play an important role in the interactions between AFIFs and both ERBB2 and RAF1. The different groups connected on 3′C and 4′-C did not have any significant effects on the interactions. This may be because the groups on glycosidic bonds have more opportunities to make contact with amino acid residues.

## 4. Material and Methods

### 4.1. Materials

AFI was obtained from Changchun, Jilin Province, China, and authenticated by Professor Jingmin Zhang, who is a voucher specimen that was deposited in Jilin University (No.20190607). Hesperidin, neohesperidin, naringin, and narirutin (Purity > 95.0% HPLC) was from Chengdu Manster Biotechnology Co., Ltd. (Chengdu, China). Agilent 1100 and Agilent Eclipse XDB-C18 column (5 μm, 4.6 × 250 mm) were applied for HPLC analysis. The aluminum adjuvant was obtained from Thermo Scientific (Rockford, IL, USA). High-glucose DMEM and TRYPSIN, 0.25% EDTA, were obtained from Gibco (Suzhou, China). FBS (Richmond, VA, USA), 4-nitrophenyl N-acetyl-β-D-glucosaminide, anti-DNP monoclonal mouse IgE, and DNP-BSA were obtained from Sigma-Aldrich (St. Louis, MO, USA). An enhanced CCK8 assay kit and Fluo 3-AM were obtained from the Tongren Institute of Chemistry (Kyushu, Japan). ELISA kits were purchased from MEIMIAN (Jiangsu, China). Ovalbumin (OVA) was obtained from Sigma-Aldrich (St. Louis, MO, USA).

### 4.2. Preparation of AFIFs

AFI was powdered and extracted by hot water three times and enriched by D-101 macroporous resin. HPLC was applied to determine the major flavonoids in the extracts named AFIFs. The extracts were separated on an Agilent Eclipse XDB-C18 column (5 μm, 4.6 × 250 mm). The mobile phase consisted of acetonitrile and water (18:82) with a flow rate of 1 mL/min at 30 °C. The detection wavelength was set at 283 nm, and the injection volume was 20 μL. The LC chromatogram of AFIFs is shown in Figure 1A. 

The standards of narirutin, hesperidin, naringin, and neohesperidin were dissolved in methanol for stock solution (concentrations of 0.060, 0.068, 0.260, and 0.240 mg/mL). All solutions were stored at 4 °C. The standards were brought to room temperature before use. 0.22 μm nylon membranes from Millipore (Burlington, MA, USA) were used to filter all the standard solutions and samples for HPLC injection.

### 4.3. Animal Experiments

Female BABL/c mice aged six weeks were provided by Changchun Institute of Biological Products Co, Ltd. (Changchun, China). All animals were housed and bred at 24 ± 2 °C under specific pathogen-free (SPF) conditions. Mice were randomly split into five groups (Control, Model group, and AFIFs (10, 50, and 100 mg/kg groups). The control group was injected with saline in a total volume of 0.2 mL on days 0, 7, and 14. Then, it was administrated with saline administered nasally on days 22 to 25. The model group was injected intraperitoneally (i.p.) with OVA (0.5 mg/mL) complex with adjuvant aluminum hydroxide in a total volume of 0.2 mL on days 0, 7, and 14. The AFIFs groups were injected intraperitoneally (i.p.) with OVA (0.5 mg/mL) complex with adjuvant aluminum hydroxide in a total volume of 0.2 mL on days 0, 7, and 14. Then, they were treated with AFIFs by intragastric gavage on days 14 to 25, one hour before nasal excitation. All groups except the control group were treated with OVA on days 22 to 25 by nasal excitation. The animals were sacrificed 24 h after the last challenge (day 25). The BWC were evaluated by the body weights of day 24 minus day 14. The rectal temperature changes were evaluated by the rectal temperature of day 24 minus day 16. The thymus and spleen indexes were calculated by the weights of the spleen and thymus divided by body weights.

Determination of antibodies and cytokines: Blood samples were collected 24 h after the last challenge and were centrifuged (5000 r/min, 4 °C) for 5 min two times. The total IgE levels was determined by ELISA. BALF was obtained and centrifuged (2500 r, 15 min, 4 °C), then the supernatant was collected for cytokine assay. IL-17A and IL-13 were determined by ELISA.

Histological assessment: Lung tissues were obtained and fixed with 10% formalin and embedded in paraffin wax. The tissue from the left lung was used for analysis. The fixed tissues were stained with H&E. Then, they were observed by an inverted microscope (OLYMPUS, TH4-200).

The name of the Ethics Committee that approved the study and all protocols were Animal Welfare and Research Ethics Committee of Jilin University. The number of the certification which verified approval of the study was SY202104001. Data sources of network pharmacology and MR analysis were derived from publicly available summary-level data, which approved by the relevant institutional revies committees.

### 4.4. Mast Cell Degranulation

Rat basophilic leukemia (RBL-2H3) cells were purchased from the cell bank of the Chinese Academy of Sciences (Shanghai, China). Mast cell degranulation was applied as illustrated before [25]. RBL-2H3 cells were sensitized with 0.5 μg/mL of anti-DNP monoclonal mouse IgE treated overnight with different concentrations of AFIFs for 2.5 h, then stimulated with DNP-BSA for 1 h, subsequently stopped with the ice about 10 min. Supernatants were collected. Cell degranulation was measured by the release of β-hex, histamine, IL-4, and intracellular Ca^2+^ concentrations of the supernatant. Intracellular Ca^2+^ concentrations were measured by the Ca^2+^ reactive fluorescence probe Fluo 3-AM.

### 4.5. Network Pharmacology Approach

Hesperidin, neohesperidin, naringin, and narirutin were used as keywords to inquire and screen the active ingredients in the Traditional Chinese Systems Pharmacology Database and Analysis Platform (TCMSP: http://www.tcmsp-e.com/#/database, accessed on 2 March 2022). The OB values of the four compounds were over 30%, and DL were more than 0.18 (Table S1). 

To ascertain the relevant targets of the four bioactive compounds in the extracts, TCMSP and STRING (http://string-db.org, accessed on 2 March 2022) were adopted. In brief, potential candidate targets were identified using the reverse pharmacodynamic profiling method. Through the retrieving process, targets were screened out after duplicates were deleted, and they were made to conform to the correct UniProt ID. Data related to allergic asthma-related targets were acquired from GeneCards (http://www.genecards.org, accessed on 2 March 2022). The keywords were “allergic asthma” and 2598 related targets were harvested jointly. To clarify the interaction between target proteins, PPI network mapping was conducted with STRING (http://string-db.org, accessed on 2 March 2022). The core targets were screened according to the degree parameter. The relationships between the targets corresponding to flavonoids and interacting protein were expressed by “component-target-disease” network and analyzed with Cytoscape 3.7.2 software. GO and KEGG enrichment analyses were conducted by using DAVID. Values of *p* < 0.05 were considered exhibiting statistical significance. The enrichment analysis was identified by the gene ratio and *p*-value.

### 4.6. Two Sample Mendelian Randomization Approach

Genome-wide association studies (GWASs) and expression quantitative trait loci studies (eQTLs) were obtained from publicly available summary-level data. Based on the results above, core targets were selected as exposure for the next studying, allergic asthma were chosen as outcome to perform the MR analysis. Genetic summary statics for allergic asthma were generated from GWAS (https://gwas.mrcieu.ac.uk, accessed on 26 February 2024) [26]. The eQTLs summary-level data were obtained from eQTLGen Consortium (https://www.eqtlgen.org/cis-eqtls.html) including 31,684 blood samples and 11 M SNPs (window size 1 Mb, MAF ≥ 1%, accessed on 26 February 2024). In this work, only cis-eQTLs were included to generate genetic instruments. The chromosome location was obtained from NCBI (https://www.ncbi.nlm.nih.gov/gene, accessed on 26 February 2024). SNPs within 100 kb windows were selected from target gene, which was associated with allergic asthma at a genome-wide significance level (*p* < 5.0 × 10^−8^). SNPs used as instruments were allowed to be in low weak linkage disequilibrium (r^2^ < 0.30) with each other. Confounding factors were confirmed by PS2 (http://www.phenoscanner.medschl.cam.ac.uk/, accessed on 26 December 2023).

### 4.7. Molecular Docking Methods

AutoDock v 4.2.6 predicts the activities of binding of proteins to flavonoids. It was integrated with AutoDock Vina and had been carefully optimized. Hesperidin, neohesperidin, naringin, and narirutin were critical compounds in flavonoids-target network and were input into AutoDock to elevate the binding activities with allergic asthma target. The PDB formats of proteins and ligand SDF formats were derived from the protein database and PubChem.

### 4.8. Statistical Analyses

Data were expressed as the mean ±SD. Statistical significance was determined by the Graph-Pad Prism 7 and SPSS 19.0 software. Statistically significant differences between the groups were determined with one-way ANOVA tests. A *p*-value of <0.05 was considered as statistically significant. 

Causal associations of AFIFs with allergic asthma was applied by summary-data-based MR approach (SMR). Inverse-variance-weighted MR (IVW-MR) was applied to combine effect estimates. R software version 4.3.2 (with TwoSample MR package) was used to conduct allele harmonization and analysis. The *F* statistic was conducted to assess the strength of SNPs used as the instrument, the SNPs with *F* > 10 to minimize weak instrument bias [27]. *F* statistics were calculated by the following formula [9,28]:

$$F=\frac{R^{2}\times (N-2)}{1-R^{2}}$$

$$R^{2}=\frac{2\times \beta^{2}\times EAF\times (1-EAF)}{2\times \beta^{2}\times EAF\times (1-EAF)+2\times SE^{2}\times N\times EAF\times (1-EAF)}$$

*R^2^* is the proportion of the variance of the trait explained by the SNP, N represents the number of participants, *EAF* represents the effect allele frequency of SNP. Β is the estimated effect of the SNP to assess its ability to uniquely predict the outcome.

The heterogeneity in dependent instruments (HEIDI) test was used to test if the observed association between gene expression and outcome was due to a linkage scenario. Cochran Q test was used to test the heterogeneity of IVW-MR method, when *p* < 0.05 means the evidence of heterogeneity [29]. MR-PRESSO analysis were applied to assess the potential horizontal pleiotropy of the SNPs when *p* < 0.05 indicates the evidence of horizontal pleiotropy. 

## 5. Conclusions

In this work, AFIFs extracted from Aurantii Fructus Immaturus, were able to alleviate allergic asthma. Our findings found hesperidin, neohesperidin, naringin, and narirutin were the main components in AFIFs, with contents of 13.21, 287.26, 322.56, and 19.35 mg/g, respectively. AFIFs effectively inhibited allergic asthma both in vivo and in vitro by inhibiting the inflammatory response and the degranulation of mast cells. Additionally, seven targets were examined for causal associations between AFIFs and allergic asthma by network pharmacology and MR analysis. Considering the pleiotropy of the SNPs, five targets (CASP3, CCND1, ICAM, ERBB2, and RAF1) were examined for their interactions with AFIFs using AutoDock Vina. The results revealed that the connection positions of glycosidic bonds play an important role in the interactions between AFIFs and both ERBB2 and RAF1. Therefore, this study helps to illustrate the impact of flavonoids on allergic asthma in theoretical and practical methods.

## Figures and Tables

**Table 1:** AutoDock Vina results of the AFIFs with targets.

	Hesperidin	Neohesperidin	Naringin	Narirutin
Vina scores (kcal/mol)				
CASP3	−9.3	−9.2	−9.5	−9.1
CCND1	−7.9	−8.4	−8.3	−7.8
ERBB2	−9.5	−8.4	−8.7	−10.1
ICAM1	−8.8	−8.5	−8.7	−8.7
RAF1	−10.0	−7.8	−8.3	−10.4

**Table 2:** Binding details of AFIFs with targets.

	Binding Sites	AA Numbers	Glu-Rha (Numbers)	Force (Numbers)
	Interaction between Flavonoids and ERBB2			
Hesperidin	13	23	5	CVHB(5), VDW(11), CHB(2), UDD(1), PDH B(1), P-Sig(1), PAlk(3)
Naringin	7	18	4	CVHB(5), VDW(11), PSig(1), Alk(1)
Narirutin	15	23	8	CVHB(5), VDW(11), CHB(4), PPT(1), Alk(1), pAlk(4)
Neohesperidin	8	20	4	CVHB(6), VDW(13), PD H B(1), PAlk(1)
	Interaction between Flavonoids and RAF1			
Hesperidin	10	22	4	CVHB(4), VDW(13), PSul(1), PPS(2), PSig(1), PAlk(2)
Naringin	5	19	1	CVHB(0), VDW(15), PSig(1), PAlk(2), U DD(2)
Narirutin	22	21	13	CVHB(9), VDW(6), CHB(1), Alk(2), PAlk(2), PSul(1), UDD(3), PDHB(2)
Neohesperidin	9	17	5	CVHB(5), VDW(9), CHB(2), PI-Ani(2)

## Data Availability

The datasets used and/or analyzed during the current study are available from the corresponding author on reasonable request.

## Supplementary Materials

The following supporting information can be downloaded at: https://www.mdpi.com/article/10.3390/ijms252413587/s1.

## Abbreviations

Aurantii Fructus Immaturus—AFI; AFI flavonoids—AFIFs; body weight changes—BWCs; bronchoalveolar lavage fluid—BALF; biological process—BP; molecular function—MF; cell composition—CC; oral bioavailability—OB; drug similarity values—DL; CVHB—conventional hydrogen bond; VDW—van der Waals; CHB—carbon hydrogen bond; UDD—unfavorable donor–donor; PDHB—pi–donor hydrogen bond; PSig—pi sigma; PAlk—pi alkyl; Alk—alkyl; PPT—pi–pi Tshaped; PSul—pi–sulfur; PAni—pi–anion; PPS—pi–pi stacked.
