# Supplementary material for: The Role of Flavonoids from Aurantii Fructus Immaturus in the Alleviation of Allergic Asthma: Theoretical and Practical Insights
Source: Int J Mol Sci. 2024 Dec 19;25(24):13587. doi: 10.3390/ijms252413587 (PMC11678185; doi:10.3390/ijms252413587)
Supplement: Supplementary file 1 [file ijms-25-13587-s001.zip › ijms-3331986-supplementary.pdf]

Table S1 Informations of AFIF and their corresponding OB and DL.

Molecule ID	Molecule name	Formula	OB (%)	DL
MOL007930	Hesperidin	$C_{28}H_{34}O_{15}$	13.33	0.67
MOL005980	Neohesperidin	$C_{28}H_{34}O_{15}$	57.44	0.27
MOL013336	Narirutin	$C_{27}H_{32}O_{14}$	8.15	0.75
MOL005812	Naringin	$C_{27}H_{32}O_{14}$	6.92	0.78

Table S2 F-statistics of SNPs.

	SNP	EA	OA	beta	eaf	chr	se	Sample size	R^2	F
BAX	rs10407758	A	C	-0.11417	0.469373	19	0.009171	23880	0.004333	103.9093
	rs10413257	C	T	-0.08392	0.286171	19	0.01035	22859	0.002042	46.77336
	rs10853805	C	A	0.058563	0.615271	19	0.009774	22103	0.001102	24.38577

rs112198199	A	C	-0.23793	0.026228	19	0.043292	10451	0.00275	28.81241
rs113360897	C	T	-0.28594	0.12902	19	0.012509	28245	0.015087	432.6168
rs11668424	A	G	-0.06557	0.368773	19	0.008337	30698	0.001372	42.16908
rs143704363	G	A	0.162278	0.045358	19	0.025992	17086	0.0021	35.94372
rs150450559	T	C	-0.16931	0.025582	19	0.029215	23292	0.001373	32.01868
rs1805419	G	A	0.075864	0.26314	19	0.014981	31459	0.000714	22.47768
rs2042250	G	A	0.156524	0.043321	19	0.026418	17280	0.001876	32.47256
rs28880605	C	T	0.057318	0.24429	19	0.009769	28374	0.000886	25.16162
rs3795052	C	A	-0.36654	0.075878	19	0.022385	26725	0.009336	251.8261
rs4645881	T	C	-0.1047	0.127623	19	0.014255	26969	0.001692	45.69766
rs4645900	T	C	-0.24455	0.030465	19	0.029395	19599	0.003335	65.56517
rs4801786	A	G	-0.10763	0.162306	19	0.019495	24432	0.001126	27.54563
rs57724609	C	T	0.144178	0.036193	19	0.027157	19850	0.001329	26.41644
rs611251	C	T	0.057896	0.153441	19	0.011127	31086	0.000691	21.50438
rs62125989	T	C	0.109141	0.296693	19	0.010729	25667	0.003012	77.54031

	rs7254332	A	C	0.107084	0.191385	19	0.01105	26451	0.002711	71.9002
	rs7255964	T	C	-0.19776	0.047771	19	0.022963	20854	0.00326	68.20388
	rs74893260	T	G	0.117106	0.093457	19	0.015581	24089	0.002003	48.33514
	rs78964204	C	A	-0.28849	0.025056	19	0.039453	13157	0.003875	51.16881
	rs8103451	A	G	0.154232	0.045665	19	0.021086	25796	0.001908	49.29823
	rs8104760	T	C	-0.17963	0.035315	19	0.025243	23040	0.002058	47.50269
CASP3	rs113437703	T	C	-0.19471	0.12872	4	0.012186	30040	0.006943	210.0077
	rs113939158	G	A	0.220907	0.105236	4	0.024144	29748	0.002661	79.35664
	rs12512933	T	C	-0.10615	0.145542	4	0.020924	31569	0.00076	24.01361
	rs13110386	A	G	0.331958	0.439617	4	0.015125	30902	0.013657	427.8428
	rs145731175	A	G	-0.1884	0.023766	4	0.033405	19318	0.001573	30.44119
	rs148800248	A	G	-0.39029	0.01669	4	0.039016	20024	0.004839	97.34854
	rs1976955	C	T	-0.07876	0.40606	4	0.008236	30570	0.002018	61.81068
	rs2001871	C	T	-0.1668	0.721017	4	0.018196	28960	0.002628	76.29579
	rs2171835	C	T	0.108752	0.74979	4	0.01703	31235	0.001176	36.76553

	rs2696051	T	G	-0.51643	0.76414	4	0.017406	31684	0.025161	817.7413
	rs3087455	G	T	0.084313	0.682859	4	0.008833	29583	0.002149	63.70418
	rs4647626	A	G	-0.34901	0.142854	4	0.011501	30902	0.023943	757.9964
	rs4647673	A	G	-0.34681	0.028145	4	0.024263	31065	0.006236	194.921
	rs62337980	T	G	0.063837	0.228705	4	0.0105	25702	0.001063	27.34705
	rs62339898	T	C	0.195499	0.044165	4	0.035911	30672	0.000942	28.93186
	rs72689210	T	C	-0.13505	0.035416	4	0.023048	27559	0.001166	32.17491
	rs72689292	T	A	-0.34231	0.05254	4	0.02316	18741	0.010602	200.7969
	rs72703555	C	T	-0.14559	0.126921	4	0.030073	31569	0.000717	22.65825
	rs75075970	T	C	0.178034	0.597161	4	0.015315	30902	0.003843	119.2038
	rs755534	G	A	-0.08729	0.597161	4	0.011376	31567	0.001498	47.36496
	rs7683512	A	G	-0.08415	0.530833	4	0.015787	30113	0.000833	25.09065
	rs965938	A	G	-0.08525	0.288346	4	0.009571	23584	0.002299	54.35171
CCND1	rs111929748	T	G	-0.18513	0.033193	11	0.024029	26992	0.002066	55.89025
	rs1385875	T	C	0.066714	0.521906	11	0.008767	31141	0.001312	40.89699

	rs17136730	C	T	0.083748	0.144248	11	0.011898	28604	0.001389	39.78283
	rs1960217	T	C	-0.18446	0.64451	11	0.00917	25974	0.010687	280.5569
	rs3212870	T	C	0.13536	0.100882	11	0.013301	31150	0.002814	87.90513
	rs518418	C	T	-0.13181	0.31168	11	0.008579	31684	0.005214	166.0663
	rs606555	C	T	-0.17057	0.863618	11	0.011578	31684	0.005545	176.6405
	rs636800	G	A	-0.08582	0.284325	11	0.008855	31346	0.00213	66.90051
	rs71465435	A	C	-0.14559	0.038686	11	0.02075	31234	0.001467	45.88772
	rs72932461	A	G	0.077435	0.631069	11	0.008275	31355	0.001905	59.85344
	rs75915166	A	C	-0.14077	0.060669	11	0.016952	30541	0.002027	62.02414
	rs79241527	T	C	-0.12484	0.037591	11	0.022896	26370	0.001051	27.75387
ERBB2	rs117866580	T	G	-0.26159	0.015055	17	0.059202	9625	0.00197	18.9945
	rs3809717	A	C	-0.03852	0.30561	17	0.008745	30603	0.000444	13.60284
	rs903506	A	G	0.559909	0.341084	17	0.008397	31268	0.097828	3390.37
	rs9915323	T	A	0.445576	0.300899	17	0.008672	31340	0.059145	1970.019
ICAM1	rs112387158	T	C	0.274221	0.058769	19	0.019331	24388	0.007435	182.6707

rs11575074	A	G	-0.13994	0.047251	19	0.021718	24302	0.001571	38.24459
rs117798643	T	C	-0.14973	0.028837	19	0.024754	29141	0.001189	34.68394
rs12720279	A	G	0.069825	0.131065	19	0.01177	31684	0.000905	28.68617
rs2278442	A	G	-0.05346	0.663225	19	0.009096	27060	0.000882	23.89839
rs2304237	C	T	0.052208	0.223934	19	0.010269	27278	0.000703	19.19207
rs281416	G	A	-0.05945	0.361226	19	0.012328	27399	0.000684	18.76271
rs281417	T	C	-0.04458	0.295929	19	0.009433	26864	0.000586	15.7514
rs281437	T	C	0.228091	0.243345	19	0.009272	27015	0.01566	429.7474
rs281440	A	G	-0.18057	0.238226	19	0.010673	24206	0.008678	211.8903
rs3093029	G	C	-0.18419	0.071445	19	0.020835	18120	0.003827	69.59748
rs35929247	A	G	0.050123	0.374431	19	0.009083	26617	0.000786	20.93792
rs5030348	G	A	0.043609	0.509551	19	0.009431	23240	0.00062	14.4186
rs7249333	A	G	0.049318	0.158694	19	0.011024	31560	0.000503	15.88182
rs74908652	C	T	0.145259	0.13619	19	0.012479	27283	0.00402	110.1217
rs75161693	C	T	-0.14804	0.072758	19	0.015377	31354	0.002605	81.8795

	rs91755	G	T	0.076203	0.499098	19	0.008009	31680	0.001915	60.78404
PEBP1	rs117105414	A	G	-0.10054	0.048423	12	0.021341	14263	0.001349	19.25804
	rs12820035	A	G	0.054653	0.150951	12	0.01228	14263	0.000948	13.53255
	rs12824153	C	A	-0.20581	0.064376	12	0.024423	13925	0.004551	63.65895
	rs142739681	A	G	-0.22072	0.033362	12	0.038747	10333	0.00295	30.56986
	rs148380960	A	C	-0.28248	0.021443	12	0.049779	8796	0.0035	30.88959
	rs149280643	C	T	0.183096	0.016055	12	0.036071	10400	0.002307	24.0439
	rs151311841	T	C	-0.32091	0.03294	12	0.036981	11486	0.006164	71.22205
	rs16948185	T	C	0.345487	0.017416	12	0.048157	12592	0.003952	49.9552
	rs1726393	C	T	-0.28274	0.222312	12	0.01425	14263	0.020518	298.7406
	rs17586342	T	G	-0.14326	0.166936	12	0.010655	14263	0.007835	112.6245
	rs2254036	A	G	0.168823	0.053839	12	0.018247	14263	0.004958	71.05616
	rs2936839	C	T	-0.06909	0.619233	12	0.00902	14255	0.00221	31.56841
	rs35506792	C	T	0.068697	0.105099	12	0.013055	14263	0.001375	19.64286
	rs35650033	C	T	-0.11257	0.154703	12	0.016377	14263	0.002626	37.54756

	rs3847650	C	T	-0.21734	0.476048	12	0.007972	14260	0.024777	362.252
	rs3924357	C	T	0.211466	0.271675	12	0.013303	14263	0.012689	183.2855
	rs55861849	G	A	0.349379	0.048339	12	0.030733	11496	0.010294	119.5475
	rs61943491	A	G	-0.23713	0.028409	12	0.026426	10333	0.006844	71.19295
	rs696345	G	C	-0.15473	0.053072	12	0.017756	13666	0.00451	61.90104
	rs7315515	C	G	0.044495	0.594925	12	0.008134	14253	0.001019	14.53602
	rs73220128	G	A	0.291117	0.137341	12	0.017191	14263	0.016251	235.5782
	rs7488596	T	C	0.129503	0.440283	12	0.008121	14263	0.008642	124.3126
	rs75514589	T	C	-0.46673	0.019294	12	0.070389	5340	0.007934	42.68829
	rs76148053	A	G	0.099332	0.038177	12	0.021617	14254	0.001288	18.37907
	rs78708372	T	C	0.213812	0.094936	12	0.013879	13100	0.012974	172.1692
RAF1	rs11708716	C	A	0.288928	0.07502	3	0.043331	31568	0.001385	43.78229
	rs11714245	G	T	0.245805	0.080075	3	0.04206	31569	0.001063	33.58633
	rs11718943	T	C	-0.10298	0.46972	3	0.019119	31231	0.000854	26.69525
	rs12636458	T	C	0.144327	0.117517	3	0.013284	27310	0.003579	98.09996

rs2454436	G	A	0.107536	0.186717	3	0.010213	31559	0.002695	85.26374
rs299641	G	A	0.082501	0.573985	3	0.008167	31567	0.002192	69.3372
rs4684871	A	G	-0.16122	0.388478	3	0.008153	31684	0.008368	267.3654
rs6442327	G	A	0.119678	0.509057	3	0.00804	31684	0.004699	149.5746
rs73022986	T	C	-0.13614	0.065386	3	0.017923	25691	0.002003	51.56072
rs7643321	G	A	0.267637	0.160153	3	0.010864	31684	0.015113	486.1492
rs9855183	T	C	-0.15562	0.096631	3	0.01352	31345	0.003598	113.1943
rs9876308	A	G	-0.13853	0.096888	3	0.013528	31234	0.002857	89.49434

Table S3 Statistical analyses of MR results

	**heterogeneity**			**pleiotropy**			**MR-PRESSO _Global**
method	Q	Q_df	Q_*p*val	egger_	se	*p*val	*p*val

					intercept			
BAX	MR Egger	14.925	23	0.897	0.0290	0.0132	0.0391	0.702
	IVW	19.714	24	0.713				
CASP3	MR Egger	21.221	20	0.384	-0.0105	0.0109	0.348	0.49
	IVW	22.202	21	0.388				
CCND1	MR Egger	9.586	10	0.478	-0.0526	0.0285	0.0950	0.313
	IVW	12.986	11	0.294				
EERB2	MR Egger	2.2639	2	0.322	-0.0012	0.0825	0.990	0.534
	IVW	2.264	3	0.519				
ICAM	MR Egger	12.072	15	0.676	0.00740	0.0194	0.709	0.766
	IVW	12.217	16	0.729				
PEBP1	MR Egger	15.808	23	0.864	-0.0458	0.0170	0.0130	0.59
	IVW	23.058	24	0.516				
RAF1	MR Egger	12.286	10	0.266	-0.0399	0.0368	0.304	0.317
	IVW	13.726	11	0.249				
